# Upregulation of TREM2 expression in M2 macrophages promotes *Brucella abortus* chronic infection

**DOI:** 10.3389/fimmu.2024.1466520

**Published:** 2024-10-21

**Authors:** Jingyu Wang, Zhirong Yan, Weiyu Zhang, Xiaofeng Liu, Jun Wang, Qisheng Peng

**Affiliations:** ^1^ State Key Laboratory for Diagnosis and Treatment of Severe Zoonotic Infectious Diseases, Key Laboratory for Zoonosis Research of the Ministry of Education, Institute of Zoonosis, and College of Veterinary Medicine, Jilin University, Changchun, China; ^2^ Institute of Microbiology Department, Jilin Provincial Center for Disease Control and Prevention, Changchun, China; ^3^ Tumor Hospital of Jilin Province, Changchun, China; ^4^ Shenzhen Center for Chronic Disease Control, Shenzhen, China

**Keywords:** *Brucella abortus*, TREM2, macrophages, chronic infection, proliferation

## Abstract

*Brucella abortus* (*B.abortus*) is a zoonotic bacterial pathogen that causes chronic host infections. The eradication of brucellosis using antibiotic therapy is often incomplete or slow. In a mouse model, the predominance of alternatively activated macrophages (also known as M2) plays an essential role in sustaining chronic infection. The underlying functional mechanism by which M2 sustains chronic infection remains unclear. Here, we show that *B. abortus* can enter M2 via triggering receptor expressed on myeloid cells 2 (TREM2) and promotes the upregulation of TREM2 expression of M2 in a type IV secretion system (T4SS)-dependent manner. Increased TREM2 enhances *B. abortus* growth within M2 by suppressing intracellular ROS production, preventing M2 pyroptosis via suppression of mitochondrial ROS (mROS), and promoting M2 proliferation by increasing β-catenin expression. In line with these results, downregulation of TREM2 expression suppressed *B. abortus* intracellular growth and M2 proliferation and induced M2 pyroptosis. In our mouse model, upregulation of TREM2 expression sustained the accumulation of M2 and *B. abortus* chronic infection, whereas downregulation of TREM2 expression restricted M2 proliferation and chronic infection. Collectively, our results suggest that targeting TREM2 may be a potential adjunct to antibiotic therapy for the prevention of chronic *Brucella* infection.

## Introduction

*Brucella abortus* is a gram-negative facultative intracellular bacterium that causes brucellosis in humans and cattle. The disease is characterized by long-term infection in the host, which leads to chronic infection. Approximately 500,000 new human cases occur annually (1). Although the World Health Organization (WHO) recommends antibiotic therapy for human brucellosis, eradication of persistent intracellular *Brucella* spp. with antibiotic treatment is often incomplete or slow (2). Strategies to enhance antibiotic therapy are hampered by our poor understanding of the mechanisms by which chronic *Brucella* infection occurs.

Interactions between different macrophage subpopulations and *Brucella* are important for understanding *Brucella* survival and disease progression. At the acute stage of *Brucella* infection, classically activated macrophage (CAM; also known as M1) subtypes predominate, and have a high bactericidal capacity and proinflammatory responses. M1 macrophages are associated with proinflammatory cytokines, including IFN-γ, TNF-α, and IL-6, and decreased bacterial survival within the spleen during the acute stage of infection (3). Conversely, *Brucella* replicates and survives preferentially in the non-inflammatory M2 phenotype in the spleen during chronic infection. Increased expression and activation of the intracellular receptor peroxisome proliferator-activated receptor γ (PPARγ), which increases intracellular glucose availability, has been identified as a causal mechanism promoting enhanced bacterial survival in M2 and the prevalence of M2 during chronic infection (4). Recently, Kerrinnes et al. reported that the intracellular availability of polyamines induced by arginase-1 expression in M2 also promotes the chronic persistence of *B. abortus* (5). However, since studies have shown that factors required for the establishment of chronic diseases *in vivo* may not necessarily depend on nutrient availability in target cells, taking advantage of host cell metabolism is likely not the sole mechanism for pathogen persistence (6, 7).

TREM2 is a cell surface receptor that belongs to the family of TREM transmembrane immunoglobulin-type receptor family. TREM2 is primarily expressed in immune cells, such as dendritic cells, microglia, macrophages, and osteoclast precursors (8), and regulates cell functions through its transmembrane binding partner DNAX-activating protein 12. The importance of TREM2 in neurodegenerative diseases has been highlighted. TREM2 has been implicated in a wide array of functions including microglial proliferation, survival, phagocytosis, and inflammatory signaling. However, TREM2 also plays important roles in regulating bacterial or viral infection, especially in models where the activation and phenotype of macrophages are key to disease progression (6, 9, 10). In a model of virus-induced chronic obstructive pulmonary disease, TREM2 expression was required for the proliferation and survival of macrophages (11). The upregulation of TREM2 expression induced by *Mycobacterium tuberculosis* infection promotes immune evasion in human macrophages (12). TREM2 deficiency promotes the activation of *Brucella*-infected macrophages, which promotes the killing of Brucella by enhancing nitric oxygen (NO) (13). Considering that M2 marophages infected with *Brucella* are more abundant during chronic infection and that TREM2 has a potential role in mediating cell proliferation and pathogen intracellular survival, we speculated that TREM2 may play a role in promoting M2 proliferation and sustaining chronic *B. abortus* infection.

## Materials and methods

### Ethics statement

The animal protocol was performed according to the regulations of the Jilin University Animal Care and Use Committee. The related animal procedures also complied with the Guide for the Care and Use of Laboratory Animals (NIH Publication No. 85-23, revised 1996).

### Antibodies and reagents

Anti-β-actin (clone: AC-15; Cat: A1978), anti-β-catenin (clone 7F7.2; Cat:04-958), DPI (Cat:4673-26-1), MitoTEMPO (cat:1334850-99-5), DCFH-DA (Cat: D6883), 3-(4,5-dimethyl-2-thiazolyl)-2,5-diphenyl-2-Htetrazolium bromide (MTT) (Cat: TOX1) and all secondary antibodies were purchased from Sigma-Aldrich (Shang Hai, China). Anti-GSDMD (clone: EPR19828; Cat: ab209845), anti-CD206(Cat: ab300621) and BrdU Cell Proliferation Assay Kit (Cat: ab287841) were purchased from Abcam (Shang Hai, China). Anti-TREM2 (D8I4C) rabbit mAb (91068) was purchased from Cell Signaling Technology (Shanghai, China). Mito-SOX (Cat: M36008) was purchased from Invitrogen (Shanghai, China). Anti-TREM2 neutralizing antibody (FAB17291A), mouse rIFN-_γ_ (Cat: NP_032363), and mouse rIL-4 (Cat: P07750) were purchased from R&D Systems (Shanghai, China). Alexa Fluor 647 Anti-Mouse F4/80 antibody (Clone: T45-2342), anti-mouse CD11b conjugated to APC-Cy7 (clone: M1/70), anti-mouse CD11c conjugated to FITC (clone: HL3), anti-murine Ly-6G conjugated to PE (clone: 1A8), and Anti-Mouse CD206 PE (Clone: Y17-505) were purchased from BD Bioscience (Shanghai, China). Other chemicals were purchased from Sigma-Aldrich (Shanghai, China) unless otherwise indicated.

### Bacterial strains and growth conditions

*B. abortus* 2308 strain was a kind gift from Dr. Ding’s laboratory (China Institute of Veterinary Drug Control, Beijing, China). *B. abortus* 2308 ΔVirB10 strain was from Dr. Chen’s laboratory (Institute of Disease Control and Prevention, AMMS Beijing, China). The green fluorescent protein (GFP)-expressing *B. abortus* strain (*Brucella*-GFP) was previously generated by our lab (14). *Brucella* strains were grown on tryptic soy agar (TSA) or tryptic soy broth (TSB) plates. The number of *B. abortus* in the cultures was calculated by comparing the OD at 600 nm with a standard curve, whereas the actual concentration of inoculation was calculated by plating on TSA plates. All live *B. abortus* manipulations were performed in BSL3 (Biosafety Level 3) facilities based on the standard operating protocols approved by the Biosafety Committee of Jilin University.

### Generation of RAW264.7 derivative cell lines

The TREM2 gene from RAW264.7-derived cDNA was obtained using PCR and then inserted into the pCDNA3.1 vector through the XhoI and ApaI restriction sites. After the plasmid was verified by DNA sequencing, pCDNA3.1-TREM2 or empty pCDNA3.1 vector was transfected into RAW264.7 cell line using Lipofectamine 3000 (Sigma), and stable lines were generated by selection with 400 μg/ml G418 (Invitrogen) (15).

TREM2 knockout plasmid LentiCRISPR-Cas9-TREM2 targeting mouse TREM2 and non-targeting control plasmid LentiCRISPR-Cas9-NT using annealed single guide RNA (sgRNA) oligonucleotide 5^’^-ACTGGTAGAGACCCGCATCA-3^’^ and 5^’^-GTACCATACCGCGTACCCTT-3^’^, respectively. sgRNAs were inserted into the LentiCRISPRv2 vector (12). The lentivirus was produced by transiently transfecting HEK293T cells using the SuperFect transfection reagent (Qiagen, USA) with three plasmid systems (LentiCRISPR-Cas9-TREM2/CRISPR-Cas9-NT, psPAX2, and pMD2.G). The virus-containing supernatant was collected 72 h after transfection, filtered through 0.45 mm filters (Millipore, USA), and stored at −80°C. For transduction, lentivirus supernatants were mixed with DEAE-dextran (5 mg/mL) (Sigma-Aldrich) for 30 min and then added to RAW264.7 cells. After 3 days of infection, cells that had stably incorporated the lentiviral construct were selected for survival in the presence of puromycin (5 μg/mL) (Santa Cruz, USA) and analyzed by Western blotting to verify the overexpression or deletion of TREM2.

### Infection of macrophages

Bone marrow-derived macrophages (BMDMs) from C57BL/6 mice were purified and cultured as previously described (16). BMDM-M1, BMDM-M2, or TREM2^−/−^ BMDM-M2 was derived from BMDM that was stimulated with 10 ng/mL of mouse rINFγor rIL-4 overnight prior to experiment and kept throughout the experiments, respectively (4). RAW-M2-NT, RAW-M2-ΔTREM2, RAW-M2-TREM2, or RAW-M2-vector were generated from RAW264.7, derivative cell lines that were stimulated with 10 ng/mL of mouse rIL-4 overnight prior to the experiment and maintained throughout the experiments (17). Cells were then infected with *B. abortus* 2308 or ΔVirB2 strains in triplicate wells of a plate at a multiplicity of infection (MOI) of 100:1 by centrifuging *B. abortus* into cells at 400*g* at 4°C for 10 min. After 15 min of infection in an atmosphere containing 5% CO_2_ at 37°C, αMEM medium was used to wash the cells three times to remove extracellular *B. abortus*, and the cells were infected for another 60 min in αMEM with 40 μg/ml gentamicin to kill extracellular *Brucella*. To investigate *Brucella* intracellular growth, infected cells were lysed with phosphate-buffered saline (PBS) with 0.1% Triton X-100 at the indicated time points, and serial dilutions of lysates were plated immediately onto TSA plates to count CFUs. When infection was performed in the presence of DPI or Mito-TEMPO, cells were pretreated for 3 h before infection. To block TREM2 signaling, the cells were pretreated with an anti-TREM2 neutralizing antibody for 20 min at room temperature before infection.

### Phagocytosis assay

RAW-M2 derivative cell lines at a density of 50,000 cells/well were infected with *Brucella*-GFP, as described above. For the opsonized samples, *B. abortus* was pretreated with 20% mouse serum for 30 min before infection. After 4 h of infection, cells were scraped from the wells. PBS was used to wash the cells three times to take dead cells. The pellet was resuspended in PBS. Phagocytosis was measured by flow cytometry through analyzing the GFP signal.

### Determination of NO production

The supernatant of each cell sample was collected at certain time points after *B. abortus* challenge. Nitric oxide (NO) content was measured by analyzing its stable product, nitrite, using Griess reagent (Sigma), as previously described (13). Data are expressed as micromoles of nitrite (mean ± SEM).

### Measurement of intracellular ROS and mitochondrial ROS

Intracellular and mitochondrial ROS levels were detected using DCFH-DA and Mito-SOX, respectively. Briefly, macrophages at a density of 1 × 10^5^ cells/well in a 24-well plate at the indicated time points were incubated with 10 μM DCFH-DA or 5 μM Mito-SOX at 37°C for 30 min in the dark. Cells were washed three times with PBS, and the fluorescence intensity was measured using Zeiss LSM 800 confocal microscopy or flow cytometry.

### LDH assay

The concentration of lactate dehydrogenase (LDH) released from the macrophages was evaluated using a commercial kit of LDH assay kit (Roche) according to the manufacturer’s procedure. In brief, supernatants of macrophages were collected at the indicated infection time points, the kit reagent was added to an aliquot of supernatant (50 μl) and incubated for 45 min, the reaction was halted, and the absorbance was analyzed at 560 nm using an ELISA plate reader. LDH released from macrophages was expressed as a percentage of total LDH activity. The total LDH was determined by lysing the cultures with Triton X-100 (13, 18).

### Viability assay

BMDM-M2 macrophage viability was measured using an MTT assay. Briefly, BMDM-M2 macrophages were cultured at a density of 2 × 10^4^ cells/well in a 96-well plate, treated with DMSO, Mito-TEMPO + Baf A1, Baf A1 or Baf A1+DPI, and then infected with *B. abortus* for 24 h. For TREM2^−/−^ BMDM-M2, M2 macrophages were cultured at a density of 2 × 10^4^ cells/well in a 96-well plate and infected with *B. abortus* for 24 h. MTT solution was added to each well at a final concentration of 0.5 mg/ml and the macrophages were incubated for another 4 h. Based on the manufacturer’s protocol, absorbance was measured at 570 nm using an automated ELISA plate reader (19).

### Primers for real-time PCR assay

For each real-time reaction, reverse-transcribed cDNA products (2 μl) were amplified by PCR in a total volume of 25 μl with 10 pmol each of the related forward and reverse primers (see **Supplementary Table**). B-actin served as the control. The fold change in mRNA levels in cells was determined using the comparative threshold cycle (Ct) method (14).

### Proliferation assay

BMDM-M2 macrophage proliferation was analyzed using the BrdU incorporation assay. Based on the protocol of the BrdU cell proliferation ELISA kit (Abcam), an assay was performed to measure macrophage proliferation. Briefly, BMDM-M2 macrophages were cultured at a density of 2 × 10^4^ cells/well in a 96-well plate and treated with DMSO, Mito-TEMPO + Baf A1, Baf A1, anti-TREM2, or isotype antibody, and then infected with *B. abortus* for 48 h in the presence of BrdU. For TREM2^−/−^ BMDM-M2, M2 macrophages were cultured at a density of 2 × 10^4^ cells/well in a 96-well plate, and then infected with *B. abortus* for 48 h in the presence of BrdU. M2 macrophages were then fixed, permeabilized, and denatured to enable the detection of BrdU incorporated with anti-BrdU antibody. After incubation with horseradish peroxidase-conjugated secondary antibody, an ELISA plate reader was used to quantify the colored reaction product at a dual wavelength of 450/550 nm. To eliminate the possibility that the results were affected by potential changes in viable cells, BrdU-positive ELISA signals were normalized to the total number of cells (19).

### Western blot

Macrophages were lysed in ice-cold RIPA (radioimmunoprecipitation) mild detergent buffer. The protein content of the cells was analyzed using a BCA protein evaluation kit (Pierce, USA). Approximately 20 mg of protein from the lysates was loaded onto SDS–PAGE and then transferred to a PVDF membrane. Membranes were incubated with the primary antibody at 4°C overnight and then washed with PBS three times at room temperature, followed by incubation with a suitable secondary antibody for 2 h at room temperature. Protein bands on the membrane were visualized using ECL reagent (GE Healthcare, Shanghai, China). The band densities of the membranes were analyzed by densitometry (model GS-700, Imaging Densitometer; Bio-Rad). The background of the visualized bands was subtracted from that of the control area (15).

### Animal experiments

Animal experiments were approved by the Institutional Animal Care and Use Committees of Jilin University (Changchun, China) and performed according to institutional guidelines. Five mice from each group (C57BL/6) were infected i.p. with 1 × 10^6^ *B. abortus*. At 3, 9, and 30 days after infection, the mice were euthanized by CO_2_ asphyxiation, and the spleens were collected aseptically at necropsy. When indicated, mice were injected intraperitoneally (i.p.) with DMSO or Baf A1 (1 mg/kg body weight daily) from 18 dpi to 30 dpi. Spleen samples were also collected for the spleen CFU assay, myeloperoxidase (MPO) activity assay, gene expression analysis, immunohistochemistry analysis, and flow cytometry (20). TREM2^−/−^ mice with C57BL/6 background were generously provided by Dr. Shuangxi Wang (Shandong University, China). Five mice from each group (wild-type or TREM2^−/−^) were infected i.p. with 1 × 10^6^ *B. abortus*. Thirty days after infection, mice were euthanized by CO_2_ asphyxiation, and the spleen was collected aseptically at necropsy for spleen CFU assay or CD11b^+^ CD206^+^ assay by flow cytometry. Serum TNFα levels were analyzed using an ELISA kit from eBioscience (Shanghai, China) according to the manufacturer’s instructions.

### Flow cytometry analysis of M2, macrophage, DC, and neutrophil in the spleen

Flow cytometry analysis for the detection of M2 macrophages, DC, and neutrophils was performed in the spleen cells of C57BL/6 or TREM2^−/−^ mice uninfected (NI) or infected with *B. abortus* for 9 or 30 days. Briefly, mice were inoculated intraperitoneally (i.p.) with 1 × 10^6^ CFU of *B. abortus* and sacrificed 9 or 30 days post-infection. The spleen cells were harvested and washed twice with sterile PBS. After washing, the cells were adjusted to 1 × 10^6^ cells in a 96-well plate, centrifuged at 1,500 rpm for 5 min at 4 ˚C and washed with PBS/BSA (PBS containing 1% bovine serum albumin). The cells were blocked with an anti-CD16 antibody for 20 min at 4 ˚C in PBS/BSA. The cells were then incubated for 20 min at 4 ˚C with a cocktail of anti-F4/80 Alexa Fluor 647, anti-CD206 PE, anti-CD11b APC-Cy7, anti-CD11c FITC, and anti- Ly-6G PE. Finally, the cells were washed twice with PBS/BSA, resuspended in PBS, and detected using Attune Acoustic Focusing equipment (Thermo Fisher Scientific). The results were analyzed using the FloWJov10 software (BD Biosciences) (18).

### Histological and immunohistochemistry

Mouse spleens were fixed with formalin, embedded in paraffin, and cut into 6-um thick sections. Spleen tissues were stained with hematoxylin–eosin (HE). The slides were analyzed for histopathology. Immunohistochemical (IHC) staining of the spleen was performed for CD206 (Abcam, Shanghai, China) according to the manufacturer’s protocols. Representative images were obtained using an Olympus microscope, and brightness was adjusted.

### Statistical analysis

All values are expressed as the means ± SD. Data were analyzed using one-way ANOVA followed by Bonferroni correction. For statistical comparisons between two groups, we used an unpaired Newman–Student t-test. All statistical analyses were performed using the SPSS software (version 10.0). Statistical significance was set at P <0.05.

## Results

### *B. abortus* infection upregulates TREM2 expression in M2

**Figure 1:**
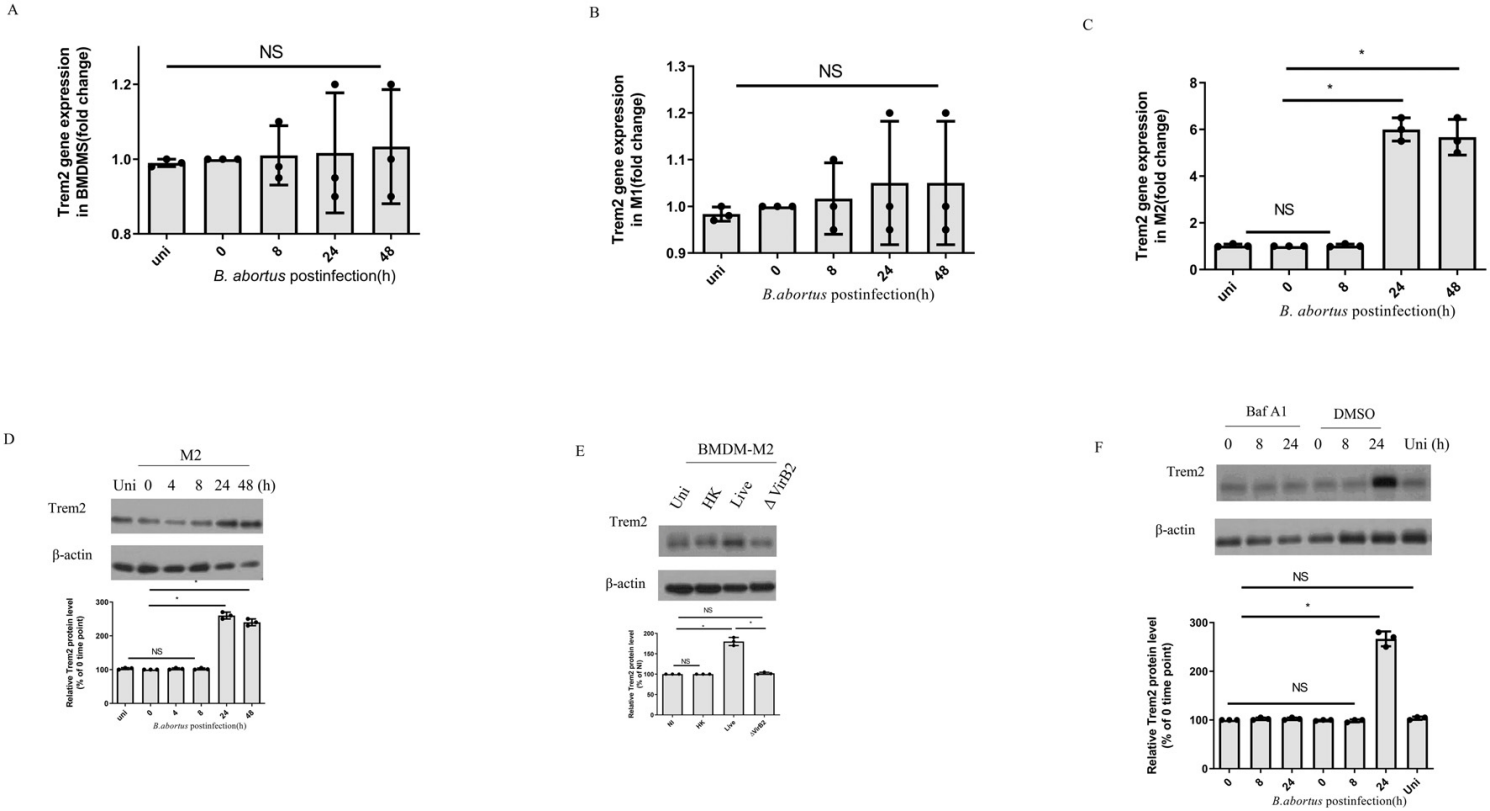
*B. abortus* infection upregulates TREM2 expression in M2. TREM2 mRNA and protein levels of *B. abortus*-infected BMDMs, BMDM-M1, or BMDM-M2 (MOI = 100) were measured by RT-PCR **(A–C)** and Western blotting **(D)** at the indicated time points, respectively. TREM2 gene expression was detected by RT-PCR with normalization to β-actin. RT-PCR data represents the result of three technical replicates (mean ± SD). **(E)** BMDM-M2 were left uninfected (NI) or infected with *B. abortus* (MOI = 100), heat-killed *B. abortus* (HK), or the ΔvirB2 mutant *B. abortus* (MOI = 100) for 24 h prior to harvesting for TREM2 protein levels assay by Western blotting. **(F)** BMDM-M2 were infected with *B. abortus* (MOI = 100) in the presence of DMSO or Baf A1(100 nM) for the indicated times. TREM2 expression in M2 were analyzed by western blotting. Immunoblots **(D–F)** were representative of three independent experiments. Detection of cellular β-actin was used as loading control. The intensity of the bands was quantified using the Bio-Rad densitometry. The 0 time point mean ratio of protein (TREM2): β-actin was defined as 100%. Quantitative data are depicted under the Western image. Western blotting and RT-PCR results were analyzed using a one-way ANOVA followed by Bonferroni correction. The uninfected (uni) cells were designed as control. P-values <0.05 were considered significant (*, P <0.05). NS indicates no significance.

Bone marrow derived macrophages (BMDMs) were infected with *B. abortus* and followed for 48 h. TREM2 mRNA levels remained unchanged in the BMDM (**Figure 1A**). BMDM polarized into M1 and infected with *B. abortus* also had similar TREM2 expression as uninfected cells (**Figure 1B**). However, BMDM polarized into M2, and infected with *B. abortus* had significantly increased TREM2 mRNA and protein levels at 24 h and 48 h after infection (**Figures 1C, D**). Moreover, upregulation of TREM2 required live bacteria, as heat-killed *B. abortus* failed to induce TREM2 expression in BMDM-M2 cells (**Figure 1E**). These data suggest that an active bacteria-driven process is required to stimulate TREM2 expression in M2 cells. As the *Brucella* effectors secreted by T4SS is involved in regulating signaling pathways of host cells (21), we examined the expression of TREM2 in BMDM-M2 infected with *B. abortus* mutant with a mutation in T4SS (deletion of the critical VirB2 subunit, ΔVirB2). We found that ΔVirB2 *B. abortus* infection failed to increase TREM2 expression in M2 (**Figure 1E**). Suppression of VirB by bafilomycin A1(Baf A1) also prevented the upregulation of TREM2 expression compared with in the DMSO control in M2 cells (**Figure 1F**). Taken together, these data show that *B. abortus* infection triggers an upregulation of TREM2 expression in M2 macrophages in a T4SS-dependent manner.

### TREM2 in M2 contributes to phagocytosis of *B. abortus*

**Figure 2:**
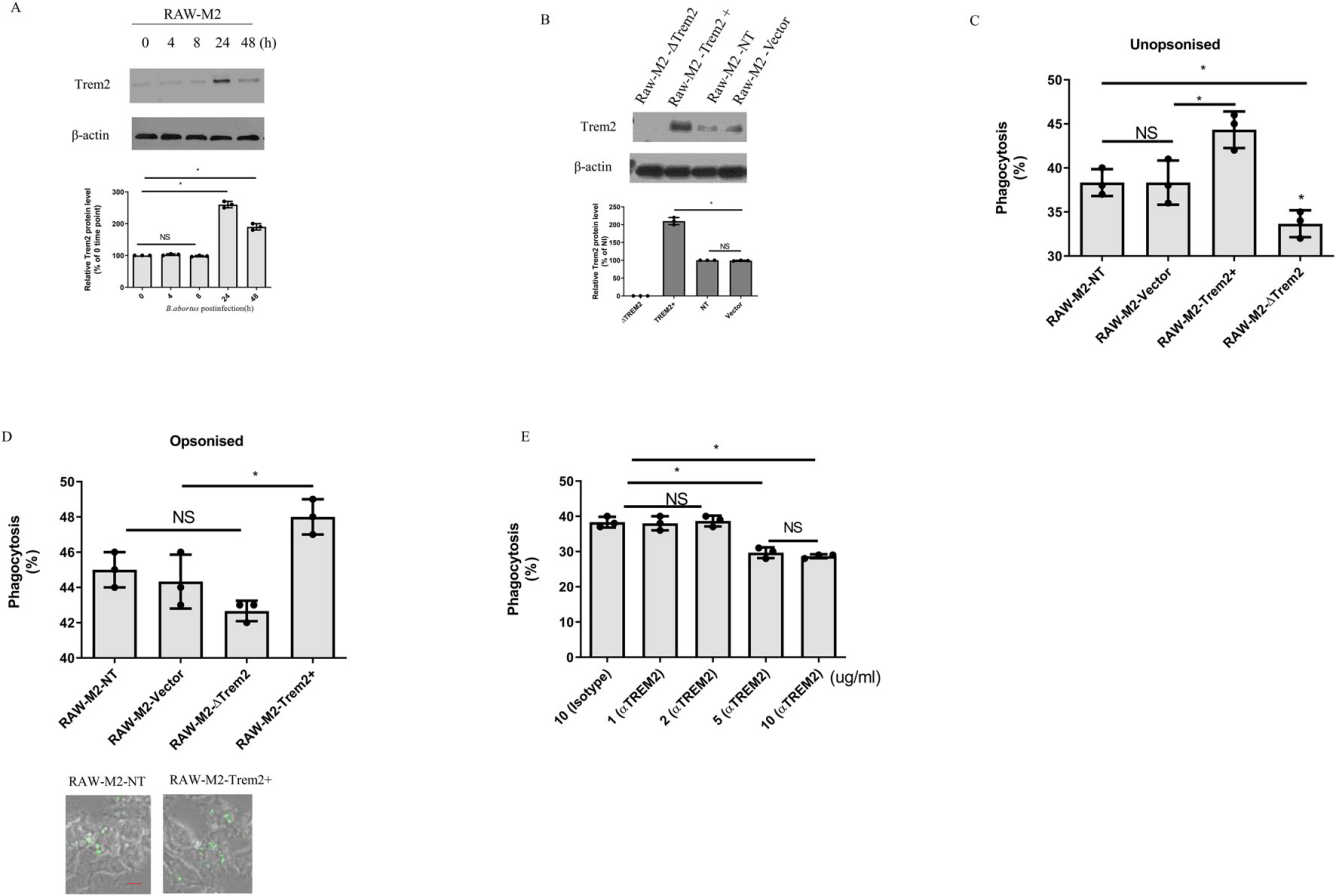
TREM2 contributes to *B. abortus* phagocytosis in M2. **(A)** TREM2 protein levels of *B. abortus*-infected RAW-M2(MOI = 100) were measured by Western blotting at the indicated time points. **(B)** RAW-M2-NT, RAW-M2-ΔTREM2, RAW-M2-Vector, or RAW-M2-TREM2+ cells were assessed for TREM2 protein expression by Western blotting. *B. abortus* (expressing GFP) were opsonized with mouse serum **(C)** or unopsonized **(D)** for 20 min, and then these bacteria were used to infect Raw-M2 derivative cell lines for another 4 h Phagocytosis was analyzed by flow cytometry through qualification of GFP^+^ cells (top panel). Representative images of phagocytosis of GFP-expressing *Brucella* in RAW-M2-NT and RAW-M2-ΔTREM2 (bottom panel) Scale bar  =  50 µm **(E)** BMDM-M2 were pretreated with TREM2 antibody (1 μg/mL to 10  μg/mL) or isotype antibody treated for 20 min, and then infected with *B. abortus* (expressing GFP) for 4 h Cells were analyzed by flow cytometry to quantify levels of phagocytosis. Immunoblots **(A, B)** were representative of three independent experiments. Detection of cellular β-actin was used as loading control. The intensity of the bands was quantified using the Bio-Rad densitometry. The 0 time point **(A)** or RAW-M2-NT **(B)** mean ratio of protein (TREM2): β-actin was defined as 100%. Quantitative data are depicted under the Western image. Phagocytosis was expressed as means ± standard deviations from two independent experiments. Phagocytosis results were analyzed using a one-way ANOVA followed by Bonferroni correction. P-values <0.05 were considered significant (*, P <0.05). NS indicates no significance.

Next, we used mouse macrophages RAW264.7 cells to investigate the role of TREM2 in M2 in mediating phagocytosis of *B. abortus* due to its functional similarity to BMDM (**Figure 2A**). We generated RAW264.7 derivative cell lines that overexpressed TREM2 (RAW-TREM2) or deleted TREM2 (RAW-ΔTREM2). For controls, RAW264.7 cells expressing endogenous TREM2 were transduced with an empty expression vector (RAW-Vector) or a vector expressing a non-target sgRNA sequence (RAW-NT). All cell lines were polarized into M2 (RAW-M2) with mouse IL-4, based on previous methods (17, 22). The overexpression or deletion of TREM2 in RAW-M2 cell lines was confirmed by western blotting (**Figure 2B**) and then infected with unopsonized GFP-expressing *B. abortus.* TREM2-deficeincy reduced the phagocytosis of *B. abortus* compared to RAW-M2-NT macrophages, whereas overexpression of TREM2 significantly increased the phagocytosis of *B. abortus* compared to RAW-M2-vector macrophages (**Figure 2C**). When *B. abortus* was opsonized, phagocytosis of *B. abortus* by TREM2 overexpression-M2 was enhanced compared to RAW-M2-vector M2, but TREM2-deficeincy did not decrease phagocytosis of *B. abortus* (**Figure 2D**), suggesting that TREM2 functions as a nonopsonic phagocytic receptor for *B. abortus*. To confirm that TREM2 contributes to phagocytosis by *B. abortus*, anti-TREM2 blocking antibody was used at various concentrations (1 ug/mL to 10 ug/mL) to treat M2 prior to infection with *B. abortus*. We found that blocking TREM2 with an anti-TREM2 antibody significantly diminished the ability of M2 to phagocytose *B. abortus* at 5 mg/mL or 10 mg/mL (**Figure 2E**). Together, our data suggest that TREM2 is involved in the phagocytosis of *B. abortus* in M2.

### *B. abortus* utilizes TREM2 to promote its intracellular survival by suppressing intracellular ROS production

**Figure 3:**
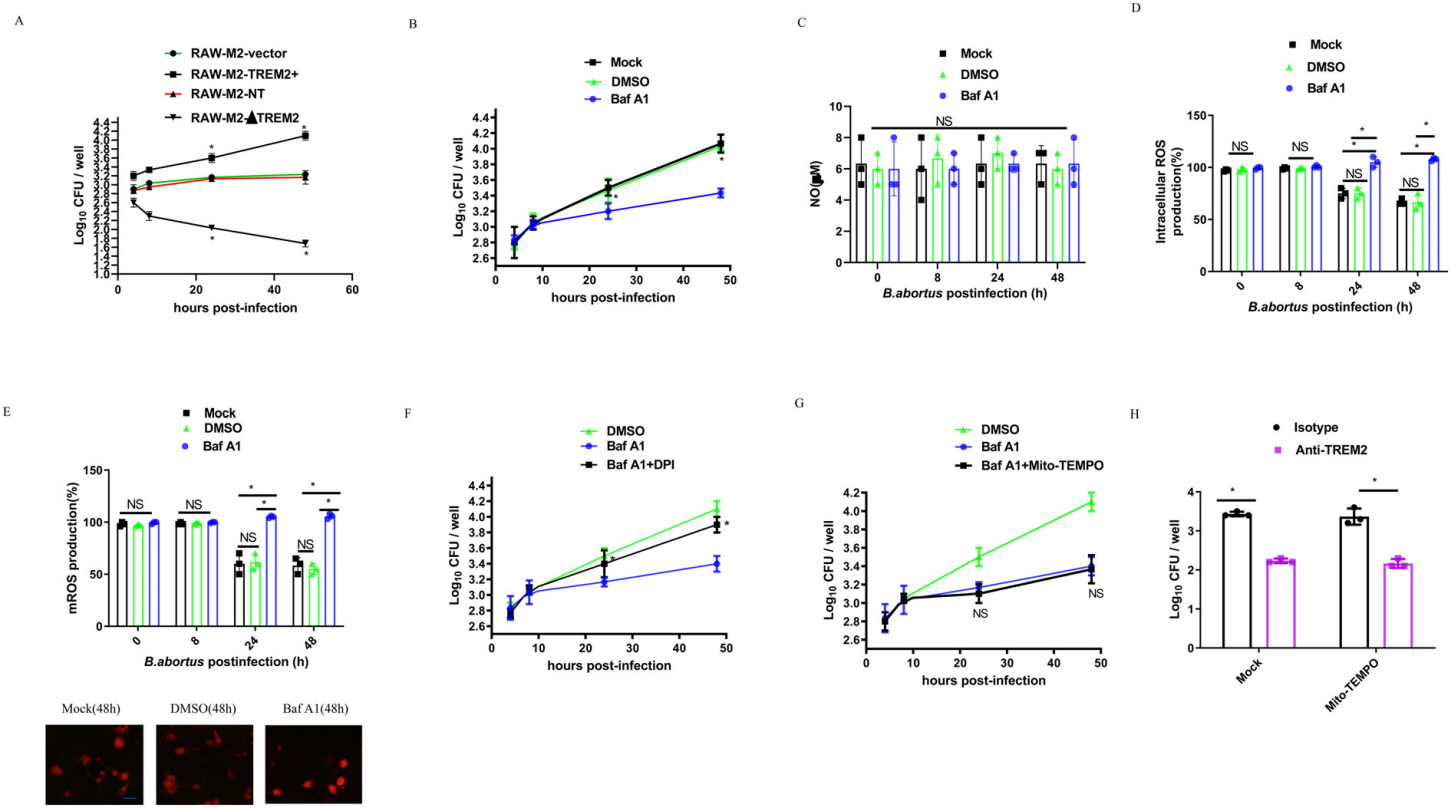
TREM2 enhances *B. abortus* survival by suppressing M2 intracellular ROS production. **(A)** RAW-M2-NT, RAW-M2-ΔTREM2, RAW-M2-Vector, or RAW-M2-TREM2+ cells were infected with *B. abortus* (MOI = 100), respectively. Intracellular numbers of *B. abortus* at the indicated times were analyzed by CFU assay. **(B)** BMDM-M2 macrophages were infected with *B. abortus* (MOI = 100) in the presence of DMSO or Baf A1 (100 nM) or mock treatment. The intracellular numbers of *B. abortus* were analyzed by CFU assay at the indicated time points. CFU was expressed as means ± standard deviations from two independent experiments. *P <0.05 vs. Baf A1. BMDM-M2 were infected with *B. abortus* (MOI = 100) for 8 h, 24 h, or 48 h in the presence of DMSO or Baf A1 (100 nM) or mock treatment. The concentration of nitric oxide (NO) in the culture supernatant collected was measured using Griess reagent **(C)**. The intracellular ROS production was detected by DCFH-DADHE. The graph demonstrates the SEM and mean of changes in production of intracellular ROS compared with the uninfected cells (as 100%) **(D)**. The mitochondria ROS (mROS) concentrations were detected by Mito-SOX. The graph demonstrates the SEM and mean of changes in production of mROS compared with the uninfected cells (as 100%) (top panel), representative images of Mito-SOX red (mitochondrial ROS marker) staining of Mock, DMSO and Baf A1 (48 h) (bottom panel). Scale bar = 50 µm **(E)**. DMSO-pretreated BMDM-M2 were infected with *B. abortus* (MOI = 100), or Baf A1 (100 nM)-pretreated BMDM-M2 were infected with *B. abortus* (MOI = 100) in the presence or absence of 10 μM DPI **(F)** or in the presence or absence of 10 μM Mito-TEMPO **(G)**, and then bacterial survival was analyzed at the indicated times by CFU assay. *P <0.05 vs. Baf A1 alone. There is no significance (NS) between Baf A1 treatment and Baf A1 plus Mito-TEMPO. **(H)** Isotype pretreated or anti-TREM2 antibody (5 ug/ml) pretreated BMDM-M2 macrophages were infected with *B. abortus* (MOI = 100) in the presence of Mock or 10 μM Mito-TEMPO, and then bacterial survival at 48 h post-infection was analyzed by CFU assay. All results were analyzed using a one-way ANOVA followed by Bonferroni correction. P-values <0.05 were considered significant (*, P <0.05). NS indicates no significance.

RAW- M2-NT, RAW-M2-ΔTREM2, RAW-M2-Vector, or RAW-M2-TREM2+ cells were infected with *B. abortus* for 48 h, and bacterial intracellular growth was determined by CFU assay. We observed that *B. abortus* survival was reduced in RAW-M2-ΔTREM2 compared to RAW- M2-NT, whereas overexpression of TREM2 increased intracellular *B. abortus* growth compared to RAW-M2-Vector (**Figure 3A**). To determine whether *B. abortus* exploits TREM2 to promote its intracellular survival, DMSO or Baf A1- treated M2, which have the same levels of TREM2 protein at 4 h post-infection (**Figure 1**), were used to determine the role of TREM2 in mediating intracellular bacterial survival during infection. As shown in **Figure 3B**, phagocytosis was similar for all conditions at 4 h post-infection, but the number of *B. abortus* in DMSO (or mock)-treated M2 was significantly increased at 24 h or 48 h post-infection compared to Baf A1- treated M2. These data indicate that upregulation of TREM2 expression is required for the increased intracellular survival of *B. abortus.*

As TREM2-mediated antibacterial defense is associated with intracellular NO or ROS production (9, 12, 13), we investigated whether NO or ROS production driven by TREM2 was responsible for the *B. abortus* survival in M2. The intracellular NO and ROS levels of DMSO or Baf A1-treated *B. abortus*-infected M2 macrophages or *B. abortus*-infected TREM2^−/−^ M2 macrophages were measured. We did not find significant changes in NO production in the experimental groups (**Figure 3C**; **Supplementary Figure S1A**), suggesting that NO was not involved in TREM2-mediated *Brucella* intracellular survival. However, M2 intracellular and mitochondrial ROS were significantly inhibited by *B. abortus* at 24 h and 48 h post-infection (**Figures 3D, E**; **Supplementary Figures S1B, C**). Baf A1-treatment or TREM2 deficiency prevented the suppression of ROS compared to mock and DMSO treated or uninfected M2 (**Figures 3D, E**; **Supplementary Figures S1B, C**). Interestingly, inhibition of cellular ROS production with the intercellular ROS inhibitor DPI (**Figure 3F**), but not mitochondrial ROS (**Figure 3G**), inhibition with Mito-TEMPO, increased the intracellular survival of *B. abortus* in M2. Using an antibody-mediated neutralization approach, we confirmed that neutralization of surface TREM2 in *B. abortus*-infected M2 cells, inhibited the intracellular survival of *B. abortus* (**Figure 3H**; **Supplementary Figure S1D**). Taken together, our data demonstrate that *B. abortus* might exploit TREM2 to suppress intracellular ROS to promote its survival within M2.

### *B. abortus* exploits TREM2 to prevents pyroptotic M2 death through reducing mitochondrial ROS production

**Figure 4:**
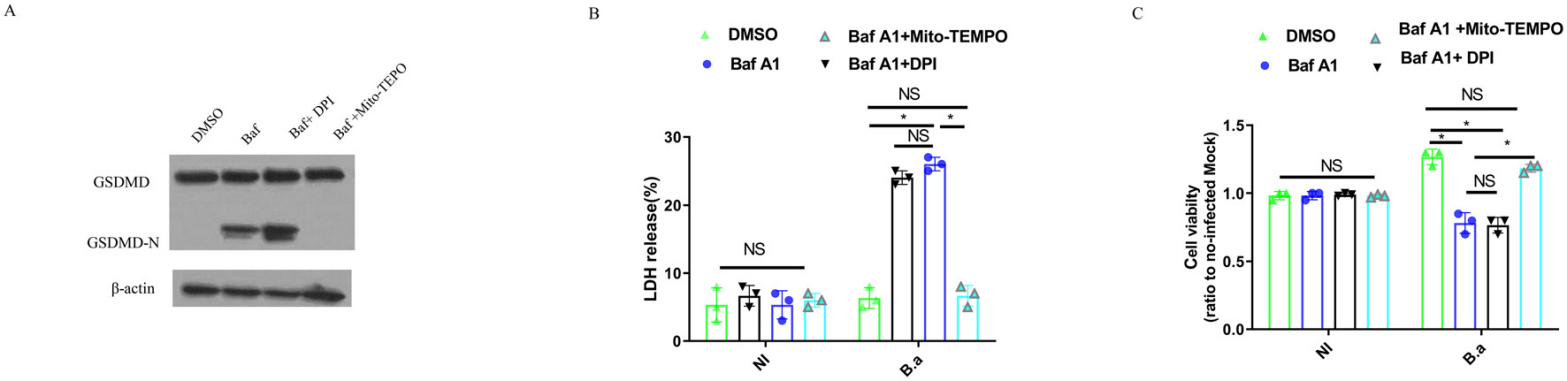
TREM2-mediated mitochondrial ROS production is required for M2 pyroptosis in response to *B. abortus*. BMDM-M2 were infected with *B. abortus*. BMDM-M2 were primed with *E. coli* LPS (1 μg/ml) for 4 h, followed by infection with opsonized *B. abortus* (MOI:100) for 24 h in the presence of DMSO, 10 μM Mito-TEMPO + Baf A1 (100 nM), 10 μM DPI + Baf A1 (100 nM) or Baf A1 (100 nM). Immunoblot showing GSDMD and GSDMD-N in lysates of BMDM-M2. Immunoblots are representative of three independent experiments **(A)**. LDH release was measured by LDH-release kit in the supernatant of cells. The graph demonstrates the SEM and mean of changes in LDH release compared with cells lysed with Triton X-100 (as 100%) **(B)**. The viability of BMDM-M2 was examined by MTT assay. The graph demonstrates the SEM and mean of changes of cell viability compared with uninfected BMDM-M2 (as 1) **(C)**. LDH and viability results were analyzed using a one-way ANOVA followed by Bonferroni correction. P-values <0.05 were considered significant t (*, P <0.05). NI means uninfected. B.a means *B. abortus*.

As overexpression of TREM2 suppressed mitochondrial ROS generation and has been shown to protect macrophages from pyroptosis during *E. coli* pulmonary infection (23), we investigated whether upregulation of TREM2 expression protects *B. abortus*-infected M2 from pyroptosis during *Brucella* infection. M2 were infected with *B. abortus* (B.a.) for 24 h in the presence of Baf A1, Baf A1 + DPI or Baf A1 + Mito-TEMPO and supernatant was harvested and submitted to western blotting (18) (**Figure 4A**). Baf A1 or Baf A1 + DPI-treated infected M2 were able to cleave GSDMD into its active GSDMD-N subunit compared to DMSO-treated infected M2. GSDMD cleavage was inhibited in Baf A1 + Mito-TEMPO-treated M2 cells (**Figure 4A**). We also found that *B. abortus* triggered a higher percentage of LDH release in Baf A1-treated M2 macrophages than in DMSO-treated M2 macrophages. Inhibition of mROS generation with Mito-TEMPO, but not intracellular ROS generation with DPI, suppress LDH release in M2 (**Figure 4B**). Consistent with GSDMD cleavage and LDH release, cell survival was suppressed significantly in Baf A1or Baf A1 + DPI-treated M2 compared to DMSO or Baf A1 + Mito-TEMPO-treated M2, as examined by MTT assay (**Figure 4C**). Using M2 macrophages from TREM2^−/−^ mice, we confirmed that TREM2 suppressed LDH release and increased cell survival by inhibiting mitochondrial ROS production (**Supplementary Figures S2A, B**). These data suggest that *B. abortus* can exploit TREM2 to prevent M2 pyroptosis by inhibiting mitochondrial ROS production.

### TREM2 promotes M2 proliferation in response to *B. abortus*

**Figure 5:**
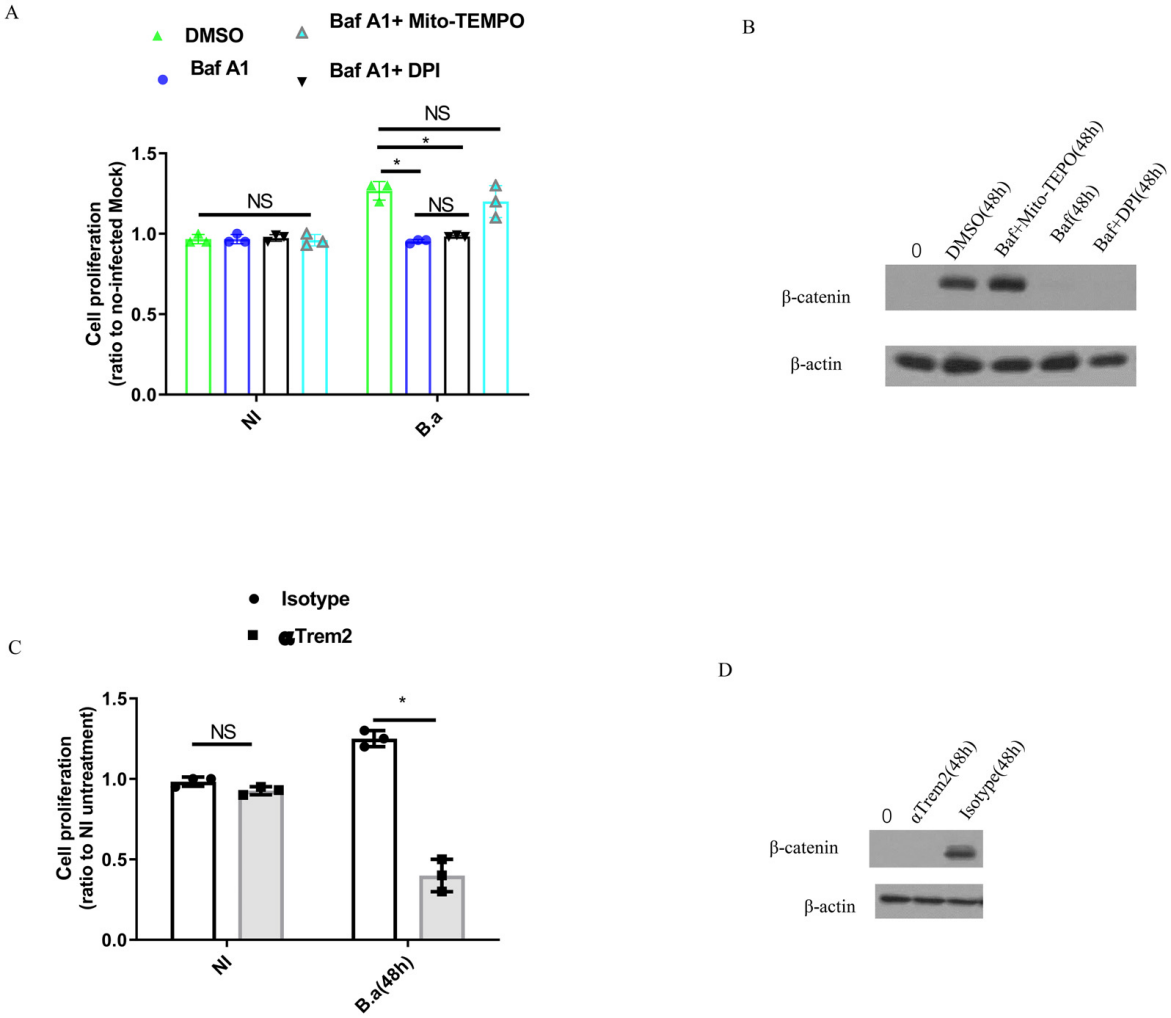
*B. abortus* induces M2 macrophages proliferation in a TREM2-dependent fashion. BMDM-M2 was uninfected (NI), or infected with *B. abortus* (MOI:100) for 48 h in the presence of DMSO, 10 μM Mito-TEMPO + Baf A1 (100 nM), or Baf A1 (100 nM), followed by using BrdU incorporation assay to measure the proliferation of BMDM-M2. The graph demonstrates the SEM and mean of changes of cell proliferation compared with uninfected BMDM-M2 (as 1) **(A)**. Immunoblot showing β-catenin in lysates of the above BMDM-M2 at 48 h post-infection **(B)**. Anti-TREM2 or isotype antibody-pretreated BMDM-M2 was uninfected (NI), or infected with *B. abortus* (MOI:100) for 48 h, followed by using BrdU incorporation assay to measure proliferation of BMDM-M2. The graph demonstrates the SEM and mean of changes of cell proliferation compared with uninfected BMDM-M2 (as 1) **(C)**. Immunoblot showing β-catenin in lysates of anti-TREM2 or isotype antibody-pretreated BMDM-M2 at 48 h post-infection **(D)**. Immunoblots are representative of three independent experiments. Cell proliferation were analyzed using a one-way ANOVA followed by Bonferroni correction. P-values <0.05 were considered significant t (*P <0.05). ns, not significant.

M2 were infected with *B. abortus* for 48 h in the presence of Baf A1, Baf A1 + DPI, DMSO or Baf A1 + Mito-TEMPO and a BrdU incorporation assay was performed to analyze M2 proliferation. Infection alone (DMSO) significantly increased M2 proliferation compared to Baf A1or Baf A1 + DPI treatment (**Figure 5A**). Interestingly, Baf A1 + Mito-TEMPO treatment also led to a significant change in M2 proliferation. The β-catenin signaling pathway is known to promote cell growth and is downstream of TREM2 (19, 24, 25). Therefore, we analyzed β-catenin expression in cell lysates from the treated cells. In line with these results, we found that infection alone (DMSO) or Baf A1 + Mito-TEMPO treatment increasedβ-catenin expression at 48 h post-infection; whereas β-catenin expression remained unchanged when *B. abortus* infected-M2 were treated with Baf A1or Baf A1 + DPI treatment (**Figure 5B**). TREM2 deficiency or blocking of TREM2 with an anti-TREM2 antibody significantly diminished M2 proliferation compared to WT cells or isotype antibody treatment (**Figure 5C**; **Supplementary Figure S3A**). Western blot data confirmed the accumulation of β-catenin in isotype antibody-treated cells or WT cells and loss of β-catenin in anti-TREM2 antibody-treated cells or TREM2^−/−^ cells (**Figure 5D**; **Supplementary Figure S3B**). Taken together, these results indicate that upregulation of TREM2 expression triggered by *B. abortus* promotes M2 proliferation by inhibiting mitochondrial ROS production, which leads to activation of the β-catenin signaling pathway.

### The upregulation of TREM2 expression leads to more abundant M2 during chronic brucellosis

**Figure 6:**
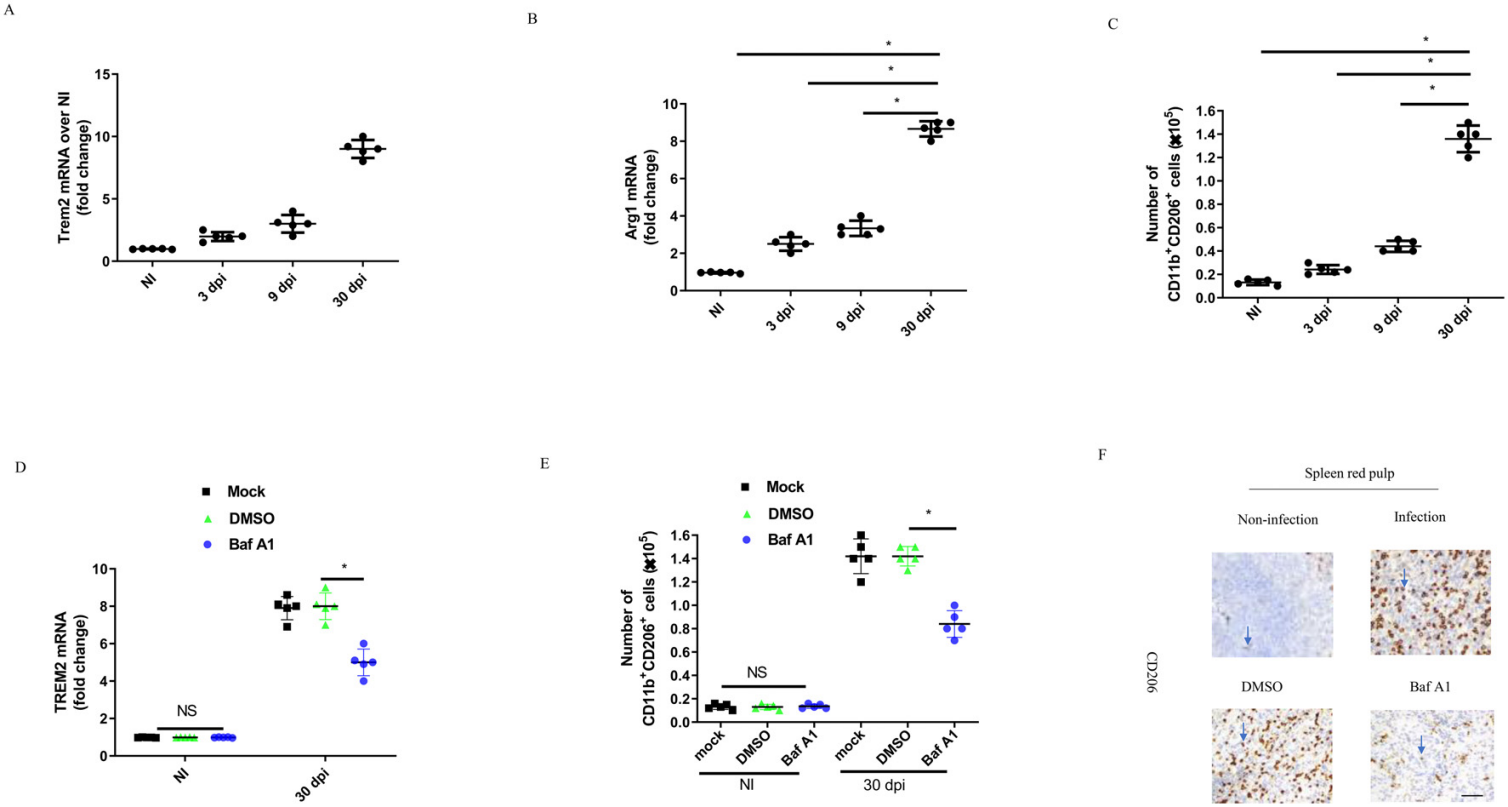
TREM2 promotes M2 proliferation during the chronic brucellosis. C57BL/6 mice were uninfected (NI) or infected intraperitoneally with 1 × 10^6^ CFU of *B. abortus.* Uninfected mice were sacrificed, and infected mice were sacrificed at 3, 9 and 30 days postinfection (dpi). RT-PCR gene expression analysis of TREM2 **(A)** or Arg1 **(B)** in CD11b^+^ splenocytes from *B. abortus*-infected or -uninfected C57BL/6 mice at the indicated times. Data are mean ± SD of five mice/group. **(C)** Spleen cells from infected C57BL/6 mice were stained for flow cytometry analysis. Cells were assessed for CD11b^+^ CD206^+^. Data are mean ± SD of five mice/group. **(D)** C57BL/6 mice were infected intraperitoneally (i.p) with 1 × 10^6^ CFU of *B. abortus*. Mice were mock treated or treated i.p. with DMSO or Baf A1 1mg per kg body weight daily from 18 dpi to 30 dpi. RT-PCR gene expression analysis of TREM2 in CD11b^+^ splenocytes from C57BL/6 mice after 30 days. Data are mean ± SD of five mice/group. **(E)** CD11b^+^ CD206^+^ number were assessed by flow cytometry analysis at NI and 30 dpi in spleen of mice. Data are mean ± SD of five mice/group. **(F)** Representative images of IHC staining of spleen red pulp with anti-CD206 antibody at 30 dpi. Data was analyzed using a one-way ANOVA followed by Bonferroni correction. P-values <0.05 were considered significant t (*P <0.05). Bar is 100 μm. ns, not significant.

To further validate the above results, we analyzed transcripts of the TREM2 and M2 marker Arg1 in splenic CD11b^+^ cells of C57BL/6 mice infected intraperitoneally with *B. abortus* for 3, 9, and 30 days. Our results showed that transcription of TREM2 was two times higher at 3 days post-infection (dpi), three times higher at 9 dpi, and eight times higher at 30 dpi, compared to uninfected conditions (**Figure 6A**). The expression level of the Arg1 gene was increased significantly at chronic infection (30 dpi) compared to acute infection (3 dpi–9 dpi) (**Figure 6B**). In line with the increase in Arg1 gene expression, *B. abortus* infection led to a significant increase in the number of CD11b^+^ CD206^+^ M2 cells in the spleen during chronic infection (**Figure 6C**). TREM2 expression was significantly decreased in splenic CD11b^+^ cells at 30 dpi when mice were injected intraperitoneally with Baf A1 (**Figure 6D**). Through flow cytometry analysis, we observed that Baf A1 treatment or TREM2 deficiency could lead to a significant decrease in CD11b^+^ CD206^+^ M2 numbers in the spleen compared to mock or DMSO treatment or wild-type mice (**Figure 6E**; **Supplementary Figure S4**). The immunohistochemistry data showed that CD206 positive cells were abundant in the spleen tissue of infected mice, but Baf A1 treatment significantly reduced CD206 positive cells at 30 dpi (**Figure 6F**). These results demonstrate that upregulation of TREM2 expression is important for sustaining abundant M2 macrophages during chronic brucellosis *in vivo*.

### TREM2 modulate *B. abortus* infection and inflammatory response during chronic brucellosis

**Figure 7:**
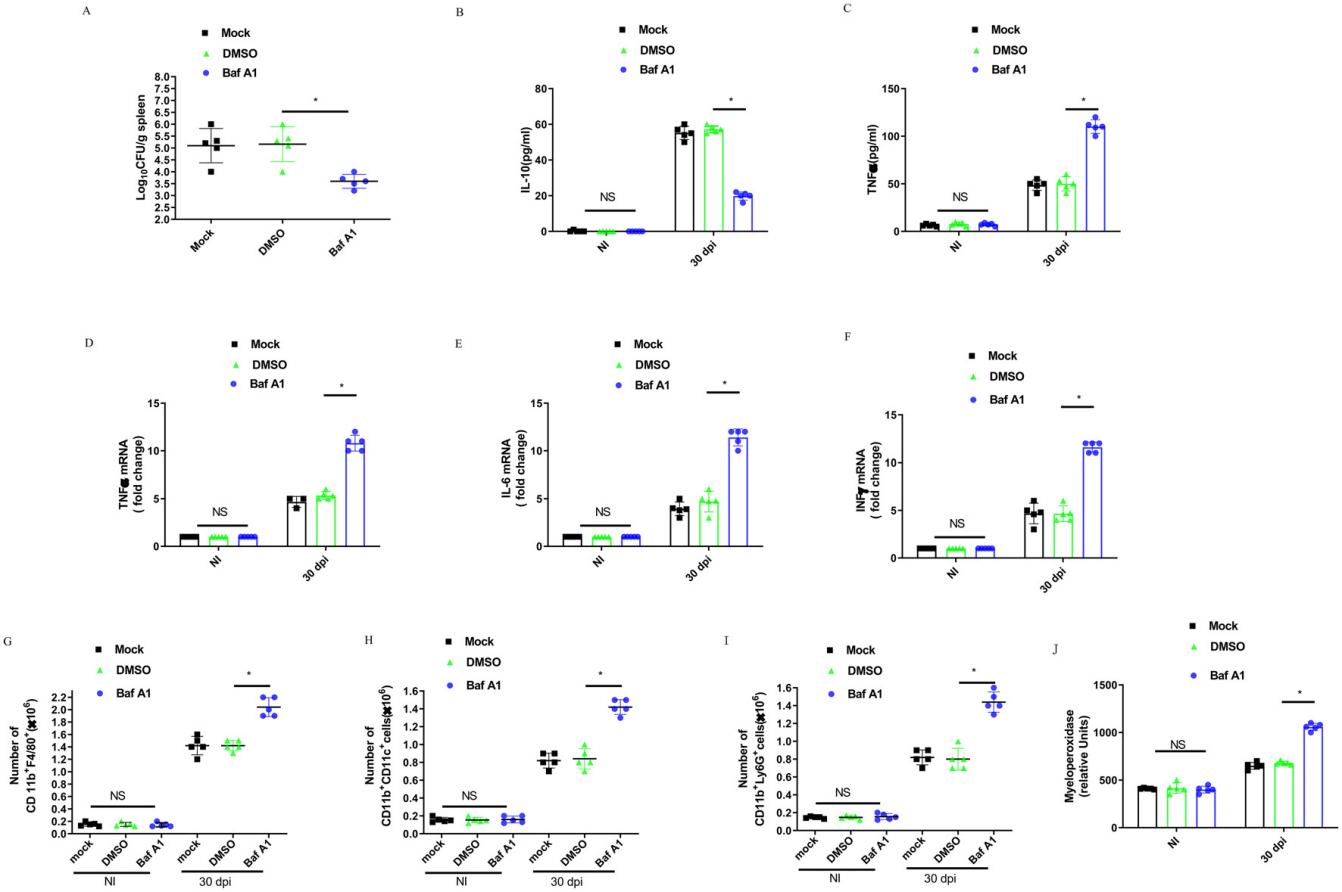
TREM2 is essential to control *B. abortus* infection and mediates inflammatory response during the chronic brucellosis. C57BL/6 mice were infected intraperitoneally with 1 × 10^6^ CFU of *B. abortus*. Mice were mock treated or treated i.p. with DMSO or Baf A1 1 mg per kg body weight daily from 18 dpi to 30 dpi. **(A)** Mice were sacrificed after 30 days, and diluted spleen homogenates were added to agar plates for CFU determination. Each symbol represents an animal and the median values are marked by horizontal bold lines. IL-10 **(B)** and TNFα **(C)** in serum were measured with ELISA kits. Data are mean ± SD of five mice/group. TNFα **(D)**, IL-6 **(E)**, and INFγ **(F)** gene expression in spleen were analyzed by RT-PCR with normalization to β-actin. Data are mean ± SD of five mice/group. Spleen cells were stained for flow cytometry analysis of CD11b^+^F4/80^+^ **(G)**, CD11b^+^ CD11c^+^ **(H)**, and CD11b^+^ Ly6G^+^ **(I)**. Splenic homogenates were submitted to a myeloperoxidase (MPO) activity assay **(J)**. Data are mean ± SD of five mice/group. Data was analyzed using a one-way ANOVA followed by Bonferroni correction. P-values <0.05 were considered significant t (*P <0.05).

C57BL/6 or TREM2^−/−^ mice were infected with *B. abortus* for 30 days, and the bacterial CFU in the spleens were evaluated. Injection of *B. abortus-*infected mice with Baf A1 or TREM2 deficiency significantly decreased bacterial numbers in the spleen compared to DMSO or mock treatment (**Figure 7A**; **Supplementary Figure S5A**). Anti-inflammatory cytokine IL-10 levels increased with infection, but significantly decreased in the serum of Baf A1 treated-mice at 30 dpi (**Figure 7B**). Treatment of *B. abortus*-infected mice with Baf A1or *B. abortus*-infected TREM2^−/−^ mice also led to a significant increase in TNF-α production compared to that in control infected mice (**Figure 7C**; **Supplementary Figure S5B**). In line with the above results, Baf A1 treated-mice also exhibited increased induction of proinflammatory cytokine transcription, including IL-6, TNF-α, and IFN-γ, in the spleen (**Figures 7D–F**).

To further evaluate the role of TREM2 in promoting *Brucella* growth within the spleen, we assessed the recruitment of immune cell populations into the spleen using flow cytometry after 30 dpi. The infected mice showed an increase in the number of macrophages (CD11b^+^F4/80^+^) (**Figure 7G**), dendritic cells (CD11b^+^CD11c^+^) (**Figure 7H**), and neutrophils (CD11b^+^LY6G^+^) (**Figure 7I**). Baf A1-treatment markedly increased these populations compared with DMSO-treated mice. Myeloperoxidase (MPO) activity in spleen cell homogenates was tested to corroborate the changes in neutrophil recruitment (**Figure 7J**). Taken together, these data suggest that TREM2 promotes anti-inflammatory M2 macrophage infiltration and proliferation, suppresses the recruitment of pro-inflammatory macrophage, dendritic cell, and neutrophil recruitment and allows chronic infection with *B. abortus.*

## Discussion

Upregulation of TREM2 expression in M2 macrophages was induced by live wild-type *B. abortus* infection but not heat-killed *Brucella*, suggesting an active bacterial-driven process rather than a simple host response to *Brucella* pathogen-associated molecular patterns. Moreover, we found that T4SS is required for the upregulation of TREM2, as the ΔvirB2 mutant or inhibition of T4SS expression suppresses infection-triggered increases in TREM2 expression. Because T4SS permits the injection of bacterial effectors inside host cells and favors bacterial intracellular growth (26), these results suggest that T4SS may contribute to TREM2 upregulation by enabling appropriate intracellular trafficking or secretion of a specific *Brucella* substrate. Interestingly, in our studies, live wild-type *B. abortus* infection was not able to induce TREM2 upregulation in M1 or other macrophages, a finding that is inconsistent with previous reports (12, 27, 28). Moreover, TREM2 was shown to mediate *Brucella*-induced NO production but not ROS production and macrophage death (13). In this study, TREM2 regulated ROS production and M2 macrophage death. The discrepancies observed in our study might be explained by the fact that the interactions between *Brucella* and macrophage subpopulations are different (3). Indeed, we have shown that only M2 macrophages exhibit increased TREM2 expression caused by *Brucella* infection.

Our previous study showed that TREM2 expression in macrophages participates in the internalization of *B. abortus* (13). In this study, we further confirmed that TREM2 expression in M2 macrophages contributes to phagocytosis of *B. abortus* through overexpression of TREM2. This was confirmed using an anti-TREM2 antibody-mediated neutralization approach. Our findings are consistent with reports that TREM2 is involved in the phagocytosis of *M*. *tuberculosis*, *E. coli*, and *S. pneumoniae* (12, 29, 30). However, increased intracellular survival of *B. abortus* in TREM2-overexpressing M2 and decreased bacterial survival in TREM2-deficient M2 are not simply attributed to initial differences in phagocytosis, because the differences in phagocytosis at 4 hpi are minimal compared to the dramatic differences in bacterial survival at 48 hpi. This suggests that TREM2 signaling plays an important role in mediating bacterial survival. Towards this end, we observed that elevated TREM2 expression participates in reducing intracellular ROS production to promote bacterial survival within M2, a finding that is reversed by the suppression of NOX2. Recently, mitochondrial ROS has also been shown to contribute to theantibacterial functions of macrophages (31, 32). However, our results showed that the inhibition of mitochondrial ROS did not increase *B. abortus* survival beyond that of untreated M2 cells. Considering that mitochondrial ROS executes its intracellular bacterial killing through the delivery of ROS into the cytoplasm (33), this phenotype could be explained by the low delivery of mitochondrial ROS into the cytoplasm, wherein the maximum levels of intracellular *B. abortus* have already been attained, and slight changes in intracellular ROS production do not lead to a detectable difference in intracellular *B. abortus* growth.

Previous studies have shown that M2 accumulation during chronic *B. abortus* infection is dependent on activation of the intracellular receptor PPARγ (4). In our experiments, we observed that TREM2 expression induced by *B. abortus* infection promoted M2 proliferation by increasing β-catenin expression. Downregulation of TREM2 expression or neutralization of surface TREM2 leads to the suppression of β-catenin-mediated proliferation. Two lines of evidence have demonstrated that TREM2 mediates M2 proliferation during chronic infection of mice. First, the increase in TREM2 expression is primarily due to the CD11b^+^ fraction, which consists predominantly of M2 during chronic infection. Secondly, Baf A1 treatment, which led to a decrease in TREM2 expression, inhibited M2 proliferation. Therefore, our data suggest that TREM2 expression is required to increase the number of splenic M2 in chronic brucellosis. Interestingly, recent evidence has demonstrated that TREM2 is involved in glucose metabolism in macrophages (34). This raises the possibility that TREM2/β-catenin signaling may intersect with the PPAR_γ_/glucose pathway to mediate M2 proliferation. However, more evidence is needed in future studies.

Collectively, we report that upregulation of TREM2 expression triggered by *B. abortus* infection contributes to the promotion of bacterial persistence and proliferation of M2 macrophages during chronic *Brucella* infection (**Supplementary Figure S6**). Considering that Brucellosis is a chronic infectious disease that ordinarily requires multiple antibiotics with frequent treatment failures and relapses, targeting TREM2 signaling through the downregulation of TREM2 expression or the use of TREM2 neutralizing antibodies could prove to be a promising adjunct to antibiotic therapy for the prevention of relapsing chronic infection.

## Data Availability

The original contributions presented in the study are included in the article/**Supplementary Material**. Further inquiries can be directed to the corresponding author.
