# Supplementary material for: Upregulation of TREM2 expression in M2 macrophages promotes Brucella abortus chronic infection
Source: Front Immunol. 2024 Oct 21;15:1466520. doi: 10.3389/fimmu.2024.1466520 (PMC11532147; doi:10.3389/fimmu.2024.1466520)
Supplement: Supplementary file 1 [file DataSheet1.pdf]

Supplementary information

Upregulation of TREM2 expression in M2 macrophages promotes Brucella abortus chronic infection

Jingyu Wang, Zhirong Yan, Weiyu Zhang, Xiaofeng Liu, Jun wang, Qisheng Peng

Inventory of Supplemental Information

Figure S1. Related to Figure 3.

Figure S2. Related to Figure 4.

Figure S3. Related to Figure 5.

Figure S4. Related to Figure 6.

Figure S5. Related to Figure 7.

Figure S6

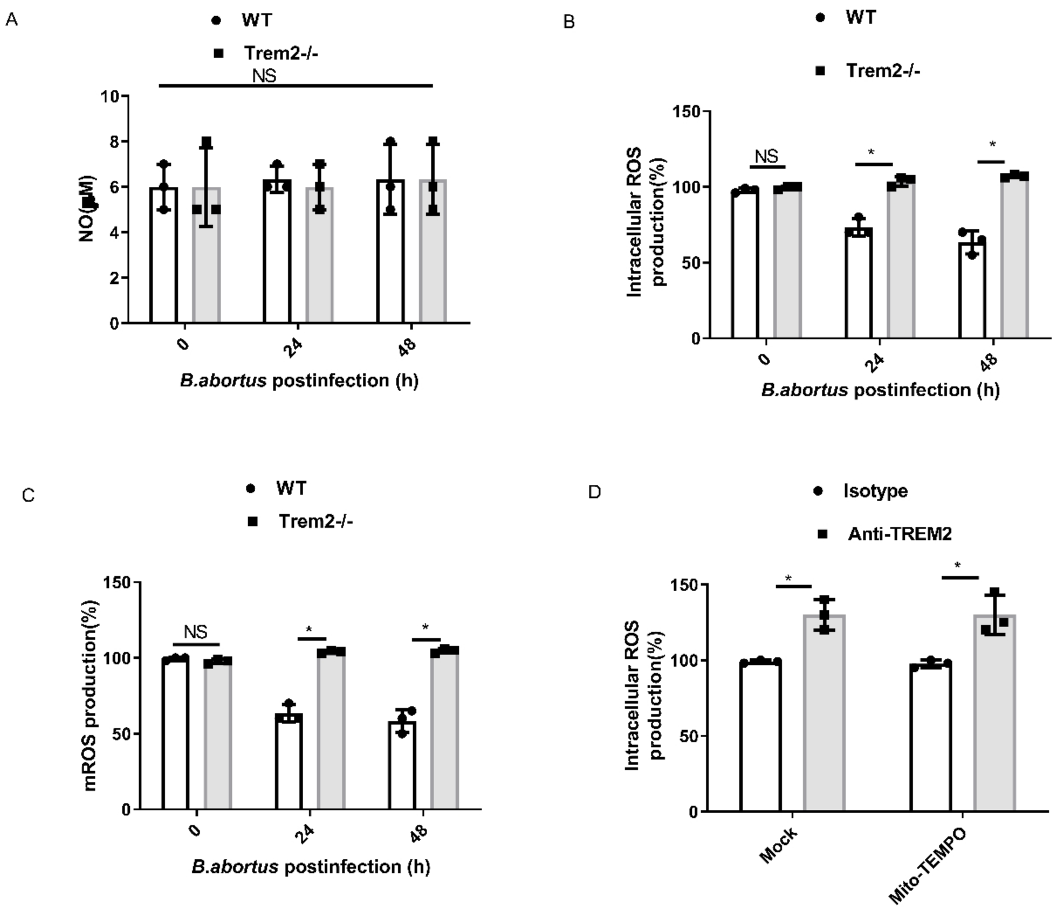

Figure S1. Related to Figure 3. BMDM-M2 from wild type (WT) or TREM2$^{-/-}$ mice were infected with *B. abortus* (MOI = 100) for 24 or 48 h. (A)The concentration of Nitric oxide (NO) in the culture supernatant collected was measured using Griess reagent. (B)The intracellular ROS production was detected by DCFH-DADHE. The graph demonstrates the SEM and mean of changes in production of intracellular ROS compared with the uninfected WT cells (as 100%). (C)The mitochondria ROS (mROS) concentrations were detected by Mito-SOX. The graph demonstrates the SEM and mean of changes in production of mROS compared with the uninfected WT cells (as 100%). (D) Isotype pretreated or anti-TREM2 antibody (5ug/ml) pretreated BMDM-M2 macrophages were

infected with B. abortus (MOI = 100) in the presence of 10 μM DPI or 10μM Mito-TEMPO, and then the intracellular ROS production was detected by DCFH-DADHE. The graph demonstrates the SEM and mean of changes in production of intracellular ROS compared with mock treatment (as 100%). The graph demonstrates the SEM and mean of changes in production of intracellular ROS compared with the uninfected WT cells (as 100%). All results were analyzed using a one-way ANOVA followed by Bonferroni correction. P values ＜0.05 were considered significant (*, P＜ 0.05). NS indicates no significance.

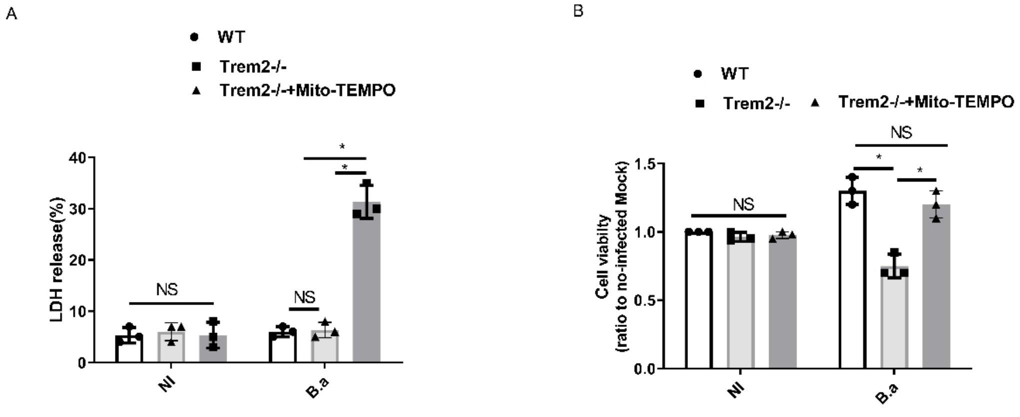

Figure S2. Related to Figure 4. BMDM-M2 from WT or TREM2$^{-/-}$ mice were left uninfected (NI) or stimulated with *B. abortus*. The cells were primed with *E. coli* LPS (1 μg/ml) for 4h, followed by infection with opsonized *B. abortus* (MOI:100) for 24h. (A) LDH release was measured by LDH-release kit in the supernatant of cells. The graph demonstrates the SEM and mean of changes in LDH release compared with cells lysed with Triton X-100 (as 100%). (B) The viability of M2 was examined by MTT assay. The graph demonstrates the SEM and mean of changes of cell viability compared with uninfected WT M2(as 1). The results were analyzed using a one-way ANOVA followed

by Bonferroni correction. P values ＜0.05 were considered significant t (*, P＜ 0.05). NI means uninfected. B.a means *B. abortus*.

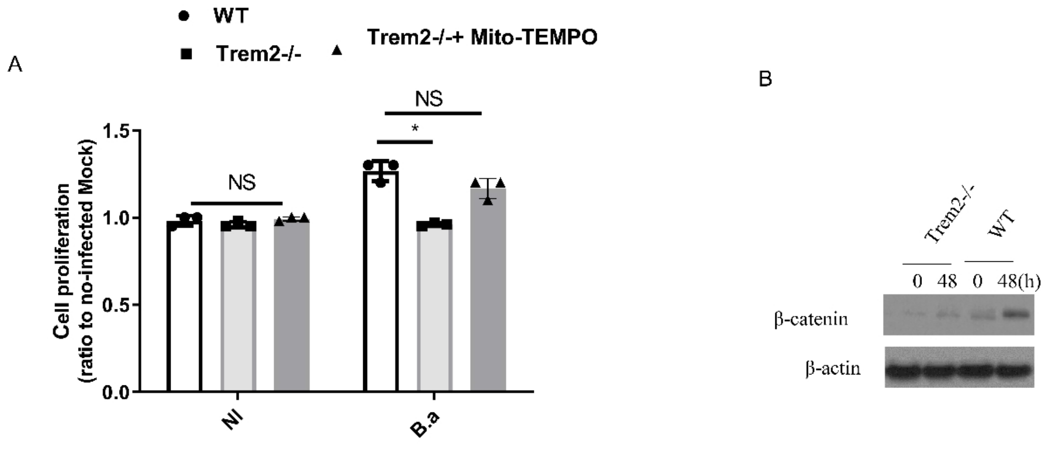

Figure S3. Related to Figure 5. BMDM-M2 from WT or TREM2$^{-/-}$ mice were uninfected (NI), or infected with *B. abortus* (MOI:100) for 48 h in the presence of 10μM Mito-TEMPO. (A) BrdU incorporation assay was used to measure the proliferation of BMDM-M2. The graph demonstrates the SEM and mean of changes of cell proliferation compared with uninfected WT BMDM-M2(as 1). (B) Immunoblot showing β-catenin in lysates of the above BMDM-M2 at 0, 48 hours postinfection. Immunoblots are representative of three independent experiments. Cells proliferation were analyzed using a one-way ANOVA followed by Bonferroni correction. P values ＜0.05 were considered significant t (* P＜ 0.05).

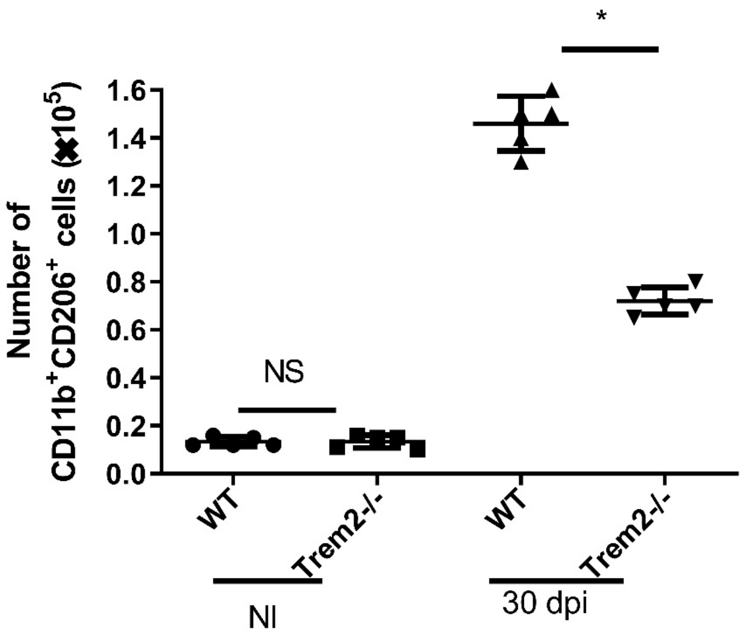

Figure S4. Related to Figure 6. WT or TREM2$^{-/-}$ mice were uninfected (NI) or infected intraperitoneally with 1 x 10^6 CFU of *B. abortus*. NI mice were sacrificed, and infected mice were sacrificed at 30 days postinfection (dpi). CD11b$^+$ CD206$^+$ number were assessed by flow cytometry analysis in spleen of mice. Data are mean ± SD of five mice/group. Data was analyzed using a one-way ANOVA followed by Bonferroni correction. P values ＜ 0.05 were considered significant t (* P＜ 0.05).

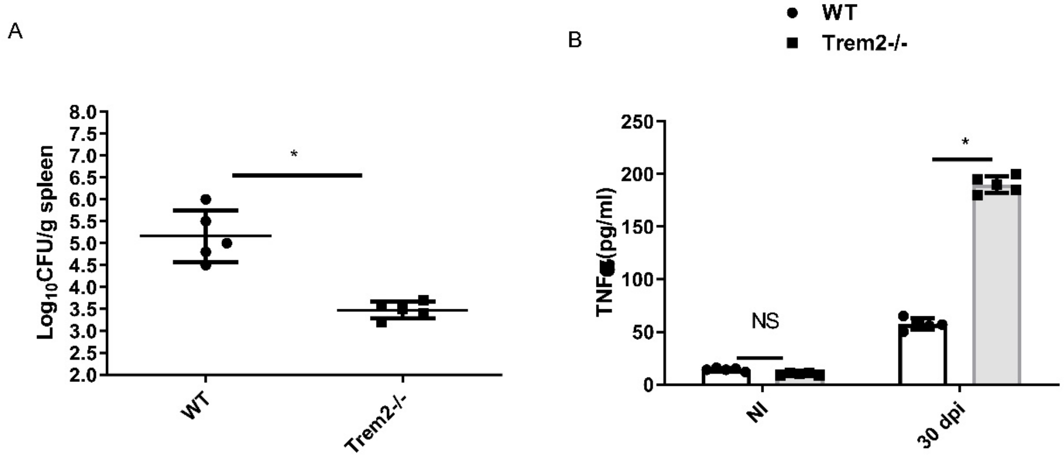

Figure S5. Related to Figure 7. WT or TREM2$^{-/-}$ mice were infected intraperitoneally with 1 x 10^6 CFU of *B. abortus* for 30 days. (A) Mice were sacrificed, and diluted spleen homogenates were added to agar plates for CFU determination. Each symbol represents an animal and the median values are marked by horizontal bold lines. (B) TNF α in serum were measured with ELISA kits. Data are mean ± SD of five mice/group. Data was analyzed using a one-way ANOVA followed by Bonferroni correction. P values ＜0.05 were considered significant t (* P＜ 0.05).

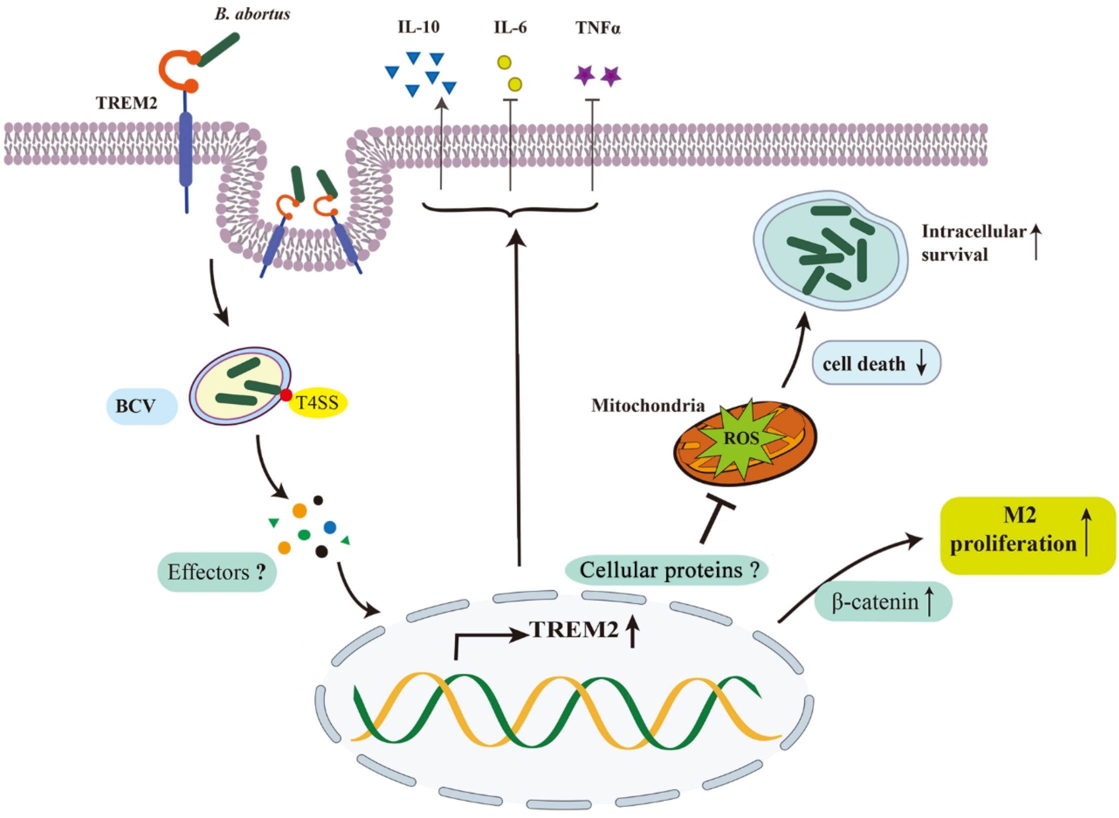

Figure s6. Summary of TREM2 in M2 defense against *Brucella* infection during the chronic infection phase. Entry of *Brucella* to Brucella -containing vacuole (BCV) via TREM2 induces a T4SS-dependent upregulation of TREM2 expression in M2, which in turn reduces M2 death by inhibiting mitochondrial ROS production. This results in the increase of intracellular bacterial growth. Upregulation of TREM2 expression also leads to the proliferation of M2 along with the decreasing the production of proinflammatory cytokines.
