# Supplementary material for: Upregulation of TREM2 expression in M2 macrophages promotes Brucella abortus chronic infection
Source: Front Immunol. 2024 Oct 21;15:1466520. doi: 10.3389/fimmu.2024.1466520 (PMC11532147; doi:10.3389/fimmu.2024.1466520)
Supplement: Supplementary file 2 [file DataSheet2.pdf]

Supplementary Table : Primers used in this study

Purpose	Sequence
mouse TREM2	TREM2-F 5’-GGCTGCTCATCTTACTCTTTG-3
	TREM2-R 5’-GAGTCATAGGGGCAAGACAC-3’
Arg1	Arg1-F 5’-AGACAGCAGAGGAGGTGAAGAGTAC-3’
	Arg1-R 5’- GGTAGTCAGTCCCTGGCTTATGGT-3’
IL-6	IL-6-F 5’-TAGTCCTTCCTACCCCAATTTCC-3’
	IL-6-R 5’-TTGGTCCTTAGCCACTCCTTC-3’
TNFα	TNFα-F 5’-AAGCCTGTAGCC CACGTCGTA-3’
	TNFα-R 5’-GGCACCACTAGTTGGTTGTCTTTG-3’
IFN-γ	IFN-γ-F 5’-CAGAGCCAAATTGTCTCCTTC-3’
	IFN-γ-R 5’-ATCCACCGGAATTTGAATCAG-3’
β -actn	β -actn-F 5’-ATTGCCGACAGGATGCAGAA-3’
	β -actn-R 5’-GCTGATCCACATC TGCTGGAA-3’
